# Methyl 2-acetonyl-4-hydr­oxy-2*H*-1,2-benzothia­zine-3-carboxyl­ate 1,1-dioxide

**DOI:** 10.1107/S1600536808019156

**Published:** 2008-07-05

**Authors:** Matloob Ahmad, Hamid Latif Siddiqui, Muhammad Zia-ur-Rehman, Graham John Tizzard, Saeed Ahmad

**Affiliations:** a Institute of Chemistry, University of The Punjab, Lahore 54590, Pakistan; b Applied Chemistry Research Centre, PCSIR Laboratories Complex, Lahore 54600, Pakistan; c School of Chemistry, University of Southampton, England; d Chemistry Department, University of Science and Technology, Bannu, Pakistan

## Abstract

In the mol­ecule of the title compound, C_13_H_13_NO_6_S, the thia­zine ring adopts a distorted sofa conformation. The enolic H atom is involved in intra­molecular O—H⋯O hydrogen bonding besides the weaker C—H⋯O=S and C—H⋯O=C inter­actions.

## Related literature

For related literature, see: Ahmad *et al.* (2008 ▶); Zia-ur-Rehman *et al.* (2005 ▶, 2006 ▶, 2007 ▶); Bihovsky *et al.* (2004 ▶); Braun (1923 ▶); Fabiola *et al.* (1998 ▶); Kojić-Prodić & Rużić-Toroš (1982 ▶); Lombardino *et al.* (1971 ▶); Turck *et al.* (1996 ▶); Weast *et al.* (1984 ▶).
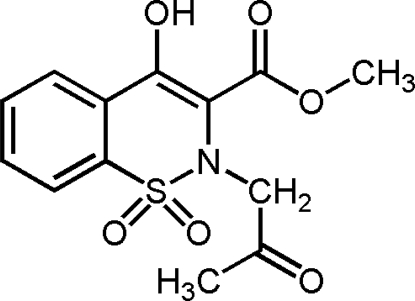


## Experimental

### 

#### Crystal data

C_13_H_13_NO_6_S
*M* *_r_* = 311.30
Orthorhombic, 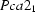

*a* = 11.7982 (3) Å
*b* = 8.7206 (2) Å
*c* = 13.2474 (4) Å
*V* = 1362.99 (6) Å^3^
*Z* = 4
Mo *K*α radiation
μ = 0.27 mm^−1^
*T* = 120 (2) K
0.50 × 0.30 × 0.15 mm

#### Data collection

Bruker–Nonius KappaCCD diffractometer
Absorption correction: multi-scan (*SADABS*; Sheldrick, 2007 ▶) *T*_min_ = 0.879, *T*_max_ = 0.961
12553 measured reflections
3095 independent reflections
2849 reflections with *I* > 2σ(*I*)
*R*_int_ = 0.041

#### Refinement

*R*[*F* ^2^ > 2σ(*F* ^2^)] = 0.040
*wR*(*F* ^2^) = 0.100
*S* = 1.13
3095 reflections
194 parameters
1 restraint
H-atom parameters constrained
Δρ_max_ = 0.57 e Å^−3^
Δρ_min_ = −0.64 e Å^−3^
Absolute structure: Flack (1983 ▶), 1466 Friedel pairs
Flack parameter: 0.00 (8)

### 

Data collection: *COLLECT* (Hooft, 1998 ▶); cell refinement: *DENZO* (Otwinowski & Minor, 1997 ▶) and *COLLECT*; data reduction: *DENZO* and *COLLECT*; program(s) used to solve structure: *SHELXS97* (Sheldrick, 2008 ▶); program(s) used to refine structure: *SHELXL97* (Sheldrick, 2008 ▶); molecular graphics: *CAMERON* (Watkin *et al.*, 1993 ▶); software used to prepare material for publication: *WinGX* (Farrugia, 1999 ▶).

## Supplementary Material

Crystal structure: contains datablocks I, global. DOI: 10.1107/S1600536808019156/bt2729sup1.cif

Structure factors: contains datablocks I. DOI: 10.1107/S1600536808019156/bt2729Isup2.hkl

Additional supplementary materials:  crystallographic information; 3D view; checkCIF report

## Figures and Tables

**Table 1:** Hydrogen-bond geometry (Å, °)

*D*—H⋯*A*	*D*—H	H⋯*A*	*D*⋯*A*	*D*—H⋯*A*
O3—H3⋯O4	0.84	1.85	2.580 (2)	145
C3—H3*A*⋯O1^i^	0.95	2.37	3.286 (3)	163
C4—H4⋯O1^ii^	0.95	2.49	3.267 (3)	139
C11—H11*A*⋯O2	0.99	2.46	2.830 (3)	102
C11—H11*B*⋯O5	0.99	2.40	2.994 (3)	118

Symmetry codes: (i) 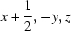; (ii) 
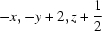
.

**Fig. 1.**
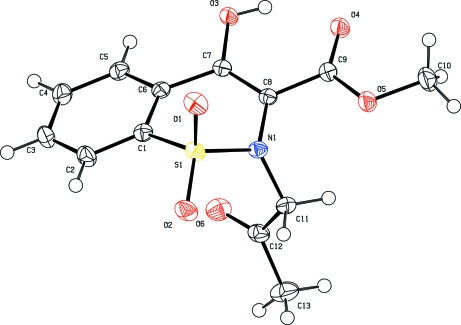
The asymmetric unit of the title compound showing the intramolecular hydrogen bond. Displacement ellipsoids are drawn at the 50% probability level.

**Fig. 2.**
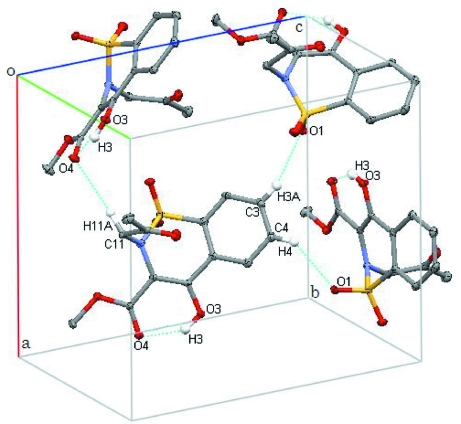
Perspective view of the three-dimensional crystal packing showing hydrogen-bonds and other intermolecular interactions (dashed lines). H atoms not involved in hydrogen bonding have been omitted for clarity.

## supplementary crystallographic information

### Comment

Benzothiazine 1,1-dioxides are known to possess a versatile range of biological activities and have been synthesized continuously since the very first synthesis in 1923 (Braun, 1923). Among these, Piroxicam (Lombardino *et al.*,1971; Zia-ur-Rehman *et al.*, 2005), and Meloxicam (Turck *et al*., 1996) are familiar for their analgesic and anti-inflammatory activities and are being used world wide as non-steroidal anti-inflammatory drugs (NSAIDs). Some of the 3,4-dihydro-1,2-benzothiazine-3-carboxylate 1,1-dioxide α-ketomide and P(2)—P(3) peptide mimetic aldehyde compounds act as potent calpain I inhibitors (Bihovsky *et al.*, 2004) while 1,2-benzothiazin-3-yl-quinazolin-4(3*H*)-ones possess antibacterial properties (Zia-ur-Rehman *et al.*, 2006). In continuation of our ongoing work (Zia-ur-Rehman *et al.*, 2007; Ahmad *et al.*, 2008), we herein report the synthesis and crystal structure of the title compound.

In this paper, the structure of the title compound (I) is reported (Scheme and figure 1). The thiazine ring, involving two double bonds, exhibits a sofa conformation; with S1/C1/C6/C7 relatively planar and N1 showing significant departure from plane due to its pyramidal geometry. The enolic hydrogen on O3 is involved in intramolecular hydrogen bonding [O3—H3···O4] with the carbonyl oxygen at C4 giving rise to a six-membered hydrogen bond ring (Table 1). The C1—S1 [1.757 (2)Å] bond is shorter than a normal C—S single bond (1.81–2.55Å) (Weast *et al.*, 1984) due to partial double bond character and is in agreement with   similar molecules (Kojić-Prodić & Ružić-Toroŝ, 1982; Fabiola *et al.*, 1998]. Each molecule is further linked to neighbouring molecules *via* weaker C—H···O=S   and C—H···O=C interactions (Table 1).

### Experimental

A mixture of mono chloroacetone (1.94 ml; 23.5 mmoles), methyl 4-hydroxy-2*H*-1,2-benzothiazine-3-carboxylate 1,1-dioxide (5.0 g,19.6 mmoles), dimethyl formamide (10.0 ml) and anhydrous sodium carbonate (4.2 g,39.2 mmoles) was stirred under nitrogen atmosphere for 3.0 h at 120 *^o^*C.The contents were then cooled to room temperature and poured over crushed ice.Title compound (I) was precipitated as white precipitates which were washed with excess of water, filtered and dried. Yield: 4.39 g; 72%; *M*.p. 455 K. Crystals were grown by slow evaporation of solution of (I) in Chloroform-Methanol (1:1) mixture.

### Refinement

All hydrogen atoms were identified in the difference map and subsequently fixed in ideal positions and treated as riding on their parent atoms. In the case of the methyl and hydroxyl H atoms the torsion angles were freely refined (three additional parameters). The following distances were used: Methyl C—H 0.98 Å. ° Methylene C—H 0.99 Å. ° Aromatic C—H 0.95 Å. ° Hydroxyl O—H 0.84 Å. U(H) was set to 1.2U_eq_ of the parent atoms or 1.5U_eq_ for methyl groups.

### Crystal data

**Table:**

C_13_H_13_NO_6_S	*F*_000_ = 648
*M**_r_* = 311.30	*D*_x_ = 1.517 Mg m^−^^3^
Orthorhombic, *P**c**a*2_1_	Mo *K*α radiation λ = 0.71073 Å
Hall symbol: P 2c -2ac	Cell parameters from 6916 reflections
*a* = 11.7982 (3) Å	θ = 2.9–27.5º
*b* = 8.7206 (2) Å	µ = 0.27 mm^−^^1^
*c* = 13.2474 (4) Å	*T* = 120 (2) K
*V* = 1362.99 (6) Å^3^	Block, colourless
*Z* = 4	0.50 × 0.30 × 0.15 mm

### Data collection

**Table:**

Bruker–Nonius KappaCCD diffractometer	3095 independent reflections
Radiation source: Bruker Nonius FR591 Rotating Anode	2849 reflections with *I* > 2σ(*I*)
Monochromator: graphite	*R*_int_ = 0.041
Detector resolution: 9.091 pixels mm^-1^	θ_max_ = 27.5º
*T* = 120(2) K	θ_min_ = 3.1º
φ and ω scans	*h* = −15→15
Absorption correction: multi-scan(SADABS; Sheldrick, 2007)	*k* = −11→11
*T*_min_ = 0.879, *T*_max_ = 0.961	*l* = −17→17
12553 measured reflections	

### Refinement

**Table:**

Refinement on *F*^2^	Hydrogen site location: inferred from neighbouring sites
Least-squares matrix: full	H-atom parameters constrained
*R*[*F*^2^ > 2σ(*F*^2^)] = 0.040	*w* = 1/[σ^2^(*F*_o_^2^) + (0.0621*P*)^2^] where *P* = (*F*_o_^2^ + 2*F*_c_^2^)/3
*wR*(*F*^2^) = 0.100	(Δ/σ)_max_ < 0.001
*S* = 1.13	Δρ_max_ = 0.57 e Å^−^^3^
3095 reflections	Δρ_min_ = −0.64 e Å^−^^3^
194 parameters	Extinction correction: SHELXL97 (Sheldrick, 2008), Fc^*^=kFc[1+0.001xFc^2^λ^3^/sin(2θ)]^-1/4^
1 restraint	Extinction coefficient: 0.067 (4)
Primary atom site location: structure-invariant direct methods	Absolute structure: Flack (1983), 1466 Friedel pairs
Secondary atom site location: difference Fourier map	Flack parameter: 0.00 (8)

### Special details

**Table:**

Experimental. *SADABS* was used to perform the Absorption correction Estimated minimum and maximum transmission: 0.6723 0.7456 The given Tmin and Tmax were generated using the *SHELX* SIZE command
Geometry. All e.s.d.'s (except the e.s.d. in the dihedral angle between two l.s. planes) are estimated using the full covariance matrix. The cell e.s.d.'s are taken into account individually in the estimation of e.s.d.'s in distances, angles and torsion angles; correlations between e.s.d.'s in cell parameters are only used when they are defined by crystal symmetry. An approximate (isotropic) treatment of cell e.s.d.'s is used for estimating e.s.d.'s involving l.s. planes.
Refinement. Refinement of *F*^2^ against ALL reflections. The weighted *R*-factor *wR* and goodness of fit *S* are based on *F*^2^, conventional *R*-factors *R* are based on *F*, with *F* set to zero for negative *F*^2^. The threshold expression of *F*^2^ > σ(*F*^2^) is used only for calculating *R*-factors(gt) *etc*. and is not relevant to the choice of reflections for refinement. *R*-factors based on *F*^2^ are statistically about twice as large as those based on *F*, and *R*- factors based on ALL data will be even larger.

### Fractional atomic coordinates and isotropic or equivalent isotropic displacement parameters (Å^2^)

**Table:**

	*x*	*y*	*z*	*U*_iso_*/*U*_eq_	
S1	0.12520 (4)	0.72682 (5)	0.71113 (5)	0.01779 (15)	
O1	0.14000 (12)	0.85887 (19)	0.64793 (13)	0.0249 (4)	
O2	0.21431 (12)	0.61542 (17)	0.71678 (14)	0.0255 (4)	
O3	−0.19773 (12)	0.91805 (17)	0.77253 (12)	0.0204 (3)	
H3	−0.2440	0.8985	0.7261	0.031*	
O4	−0.27020 (12)	0.78295 (17)	0.61171 (13)	0.0229 (4)	
O5	−0.15104 (13)	0.62248 (18)	0.53092 (12)	0.0234 (4)	
O6	−0.03227 (14)	0.48788 (19)	0.85565 (13)	0.0287 (4)	
N1	0.00988 (15)	0.63873 (19)	0.67500 (15)	0.0172 (4)	
C1	0.08889 (18)	0.7943 (2)	0.83187 (17)	0.0173 (4)	
C2	0.16859 (19)	0.8042 (3)	0.90839 (18)	0.0231 (5)	
H2	0.2436	0.7675	0.8984	0.028*	
C3	0.1371 (2)	0.8685 (3)	0.9994 (2)	0.0258 (5)	
H3A	0.1909	0.8765	1.0525	0.031*	
C4	0.0269 (2)	0.9218 (3)	1.01423 (18)	0.0244 (5)	
H4	0.0061	0.9659	1.0771	0.029*	
C5	−0.05253 (19)	0.9105 (2)	0.93717 (17)	0.0195 (4)	
H5	−0.1274	0.9473	0.9476	0.023*	
C6	−0.02314 (17)	0.8457 (2)	0.84476 (17)	0.0169 (4)	
C7	−0.10643 (17)	0.8267 (2)	0.76334 (17)	0.0156 (4)	
C8	−0.09225 (18)	0.7266 (2)	0.68505 (15)	0.0155 (4)	
C9	−0.17931 (18)	0.7142 (2)	0.60656 (16)	0.0172 (4)	
C10	−0.2356 (2)	0.6047 (3)	0.4522 (2)	0.0327 (6)	
H10A	−0.3074	0.5716	0.4822	0.049*	
H10B	−0.2098	0.5275	0.4035	0.049*	
H10C	−0.2465	0.7029	0.4176	0.049*	
C11	0.00404 (19)	0.4701 (2)	0.67907 (18)	0.0202 (4)	
H11A	0.0763	0.4277	0.6532	0.024*	
H11B	−0.0572	0.4350	0.6335	0.024*	
C12	−0.01724 (18)	0.4058 (2)	0.78301 (19)	0.0216 (5)	
C13	−0.0175 (2)	0.2338 (3)	0.7899 (2)	0.0329 (6)	
H13A	−0.0154	0.2028	0.8609	0.049*	
H13B	0.0492	0.1928	0.7549	0.049*	
H13C	−0.0864	0.1935	0.7582	0.049*	

### Atomic displacement parameters (Å^2^)

**Table:**

	*U*^11^	*U*^22^	*U*^33^	*U*^12^	*U*^13^	*U*^23^
S1	0.0125 (2)	0.0212 (2)	0.0196 (2)	0.00081 (16)	0.0010 (2)	0.0031 (3)
O1	0.0198 (8)	0.0285 (8)	0.0263 (10)	−0.0038 (6)	0.0031 (6)	0.0092 (7)
O2	0.0170 (7)	0.0302 (8)	0.0293 (9)	0.0066 (5)	−0.0005 (7)	−0.0005 (8)
O3	0.0159 (7)	0.0222 (7)	0.0232 (8)	0.0050 (5)	−0.0035 (6)	−0.0042 (7)
O4	0.0176 (8)	0.0256 (7)	0.0256 (8)	0.0052 (6)	−0.0034 (6)	−0.0057 (7)
O5	0.0237 (9)	0.0267 (9)	0.0198 (8)	0.0058 (6)	−0.0048 (6)	−0.0056 (7)
O6	0.0361 (10)	0.0257 (8)	0.0243 (9)	0.0012 (7)	0.0043 (7)	0.0048 (7)
N1	0.0148 (9)	0.0162 (7)	0.0205 (9)	0.0014 (6)	0.0018 (7)	0.0003 (7)
C1	0.0173 (10)	0.0157 (8)	0.0190 (10)	−0.0019 (7)	0.0016 (9)	0.0019 (9)
C2	0.0164 (11)	0.0253 (10)	0.0276 (12)	−0.0014 (8)	−0.0053 (9)	0.0016 (10)
C3	0.0267 (13)	0.0267 (12)	0.0241 (12)	−0.0015 (9)	−0.0110 (9)	0.0007 (10)
C4	0.0310 (13)	0.0232 (10)	0.0191 (11)	0.0003 (9)	−0.0031 (9)	−0.0014 (9)
C5	0.0205 (11)	0.0158 (9)	0.0222 (10)	−0.0019 (7)	0.0009 (9)	0.0011 (9)
C6	0.0181 (11)	0.0119 (8)	0.0208 (11)	−0.0029 (7)	−0.0013 (8)	0.0036 (8)
C7	0.0129 (9)	0.0150 (9)	0.0188 (10)	−0.0008 (7)	0.0016 (8)	0.0030 (8)
C8	0.0137 (9)	0.0141 (9)	0.0188 (11)	−0.0009 (7)	0.0004 (8)	0.0021 (8)
C9	0.0161 (10)	0.0187 (9)	0.0168 (10)	−0.0009 (8)	0.0005 (8)	0.0004 (8)
C10	0.0323 (14)	0.0408 (12)	0.0250 (12)	0.0121 (11)	−0.0117 (10)	−0.0120 (12)
C11	0.0205 (10)	0.0168 (9)	0.0234 (11)	0.0035 (8)	−0.0005 (8)	−0.0019 (9)
C12	0.0144 (11)	0.0200 (10)	0.0303 (13)	0.0005 (7)	−0.0008 (9)	0.0035 (10)
C13	0.0332 (15)	0.0194 (11)	0.0460 (17)	0.0012 (9)	0.0037 (12)	0.0042 (11)

### Geometric parameters (Å, °)

**Table:**

S1—O2	1.4335 (14)	C4—C5	1.389 (3)
S1—O1	1.4345 (17)	C4—H4	0.9500
S1—N1	1.6341 (19)	C5—C6	1.392 (3)
S1—C1	1.757 (2)	C5—H5	0.9500
O3—C7	1.345 (2)	C6—C7	1.469 (3)
O3—H3	0.8400	C7—C8	1.366 (3)
O4—C9	1.231 (3)	C8—C9	1.465 (3)
O5—C9	1.325 (3)	C10—H10A	0.9800
O5—C10	1.452 (3)	C10—H10B	0.9800
O6—C12	1.212 (3)	C10—H10C	0.9800
N1—C8	1.434 (3)	C11—C12	1.508 (3)
N1—C11	1.473 (3)	C11—H11A	0.9900
C1—C2	1.385 (3)	C11—H11B	0.9900
C1—C6	1.406 (3)	C12—C13	1.503 (3)
C2—C3	1.381 (4)	C13—H13A	0.9800
C2—H2	0.9500	C13—H13B	0.9800
C3—C4	1.394 (4)	C13—H13C	0.9800
C3—H3A	0.9500		
			
O2—S1—O1	119.03 (10)	O3—C7—C6	113.72 (18)
O2—S1—N1	107.90 (9)	C8—C7—C6	123.24 (18)
O1—S1—N1	107.93 (10)	C7—C8—N1	120.98 (18)
O2—S1—C1	110.99 (11)	C7—C8—C9	119.99 (18)
O1—S1—C1	106.99 (10)	N1—C8—C9	118.93 (18)
N1—S1—C1	102.77 (10)	O4—C9—O5	123.7 (2)
C7—O3—H3	109.5	O4—C9—C8	122.39 (19)
C9—O5—C10	115.80 (17)	O5—C9—C8	113.86 (18)
C8—N1—C11	119.38 (17)	O5—C10—H10A	109.5
C8—N1—S1	114.92 (13)	O5—C10—H10B	109.5
C11—N1—S1	119.83 (15)	H10A—C10—H10B	109.5
C2—C1—C6	121.9 (2)	O5—C10—H10C	109.5
C2—C1—S1	121.44 (17)	H10A—C10—H10C	109.5
C6—C1—S1	116.52 (16)	H10B—C10—H10C	109.5
C3—C2—C1	118.8 (2)	N1—C11—C12	114.36 (18)
C3—C2—H2	120.6	N1—C11—H11A	108.7
C1—C2—H2	120.6	C12—C11—H11A	108.7
C2—C3—C4	120.6 (2)	N1—C11—H11B	108.7
C2—C3—H3A	119.7	C12—C11—H11B	108.7
C4—C3—H3A	119.7	H11A—C11—H11B	107.6
C5—C4—C3	120.1 (2)	O6—C12—C13	122.8 (2)
C5—C4—H4	119.9	O6—C12—C11	121.97 (18)
C3—C4—H4	119.9	C13—C12—C11	115.3 (2)
C6—C5—C4	120.4 (2)	C12—C13—H13A	109.5
C6—C5—H5	119.8	C12—C13—H13B	109.5
C4—C5—H5	119.8	H13A—C13—H13B	109.5
C5—C6—C1	118.06 (19)	C12—C13—H13C	109.5
C5—C6—C7	121.68 (19)	H13A—C13—H13C	109.5
C1—C6—C7	120.2 (2)	H13B—C13—H13C	109.5
O3—C7—C8	123.04 (19)		
			
O2—S1—N1—C8	167.94 (15)	C5—C6—C7—O3	20.3 (3)
O1—S1—N1—C8	−62.23 (17)	C1—C6—C7—O3	−161.46 (18)
C1—S1—N1—C8	50.61 (17)	C5—C6—C7—C8	−160.21 (19)
O2—S1—N1—C11	15.1 (2)	C1—C6—C7—C8	18.0 (3)
O1—S1—N1—C11	144.91 (16)	O3—C7—C8—N1	175.91 (18)
C1—S1—N1—C11	−102.25 (17)	C6—C7—C8—N1	−3.5 (3)
O2—S1—C1—C2	31.3 (2)	O3—C7—C8—C9	−0.6 (3)
O1—S1—C1—C2	−100.01 (18)	C6—C7—C8—C9	−179.97 (18)
N1—S1—C1—C2	146.46 (18)	C11—N1—C8—C7	118.1 (2)
O2—S1—C1—C6	−152.25 (15)	S1—N1—C8—C7	−34.9 (2)
O1—S1—C1—C6	76.41 (16)	C11—N1—C8—C9	−65.4 (3)
N1—S1—C1—C6	−37.13 (17)	S1—N1—C8—C9	141.61 (16)
C6—C1—C2—C3	−0.9 (3)	C10—O5—C9—O4	−0.7 (3)
S1—C1—C2—C3	175.34 (17)	C10—O5—C9—C8	179.00 (18)
C1—C2—C3—C4	0.3 (3)	C7—C8—C9—O4	−5.5 (3)
C2—C3—C4—C5	0.1 (3)	N1—C8—C9—O4	177.96 (18)
C3—C4—C5—C6	0.2 (3)	C7—C8—C9—O5	174.79 (18)
C4—C5—C6—C1	−0.7 (3)	N1—C8—C9—O5	−1.8 (3)
C4—C5—C6—C7	177.53 (19)	C8—N1—C11—C12	−73.3 (3)
C2—C1—C6—C5	1.1 (3)	S1—N1—C11—C12	78.4 (2)
S1—C1—C6—C5	−175.30 (15)	N1—C11—C12—O6	2.6 (3)
C2—C1—C6—C7	−177.19 (19)	N1—C11—C12—C13	−176.96 (19)
S1—C1—C6—C7	6.4 (2)		

### Hydrogen-bond geometry (Å, °)

**Table:**

*D*—H···*A*	*D*—H	H···*A*	*D*···*A*	*D*—H···*A*
O3—H3···O4	0.84	1.85	2.580 (2)	145
C3—H3A···O1^i^	0.95	2.37	3.286 (3)	163
C4—H4···O1^ii^	0.95	2.49	3.267 (3)	139
C11—H11A···O2	0.99	2.46	2.830 (3)	102
C11—H11B···O5	0.99	2.40	2.994 (3)	118

Symmetry codes: (i) *x*+1/2, −*y*, *z*; (ii) −*x*, −*y*+2, *z*+1/2.
